# Effective dose conversion coefficients for radionuclides exponentially distributed in the ground

**DOI:** 10.1007/s00411-012-0432-y

**Published:** 2012-08-07

**Authors:** Kimiaki Saito, Nobuhito Ishigure, Nina Petoussi-Henss, Helmut Schlattl

**Affiliations:** 1 Japan Atomic Energy Agency, Uchisaiwai-cho, Chiyoda-ku, Tokyo 100-8577 Japan; 2 Graduate School of Medicine, Nagoya University, 1-1-20 Daiko-Minami, Higashi-ku, Nagoya City, Aichi Prefecture 461-8673 Japan; 3 Department of Radiation Physics and Diagnostics, Helmholtz Zentrum München-German Research Center for Environmental Health, Ingolstädter Landstr. 1, 85764 Neuherberg, Germany

**Keywords:** Effective dose, Conversion coefficients, Ground source, Exponential distribution, Radionuclides

## Abstract

In order to provide fundamental data required for dose evaluation due to environmental exposures, effective dose conversion coefficients, that is, the effective dose rate per unit activity per unit area, were calculated for a number of potentially important radionuclides, assuming an exponential distribution in ground, over a wide range of relaxation depths. The conversion coefficients were calculated for adults and a new-born baby on the basis of dosimetric methods that the authors and related researchers have previously developed, using Monte Carlo simulations and anthropomorphic computational phantoms. The differences in effective dose conversion coefficients due to body size between the adult and baby phantoms were found to lie within 50 %, for most cases; however, for some low energies, differences could amount to a factor of 3. The effective dose per unit source intensity per area was found to decrease by a factor of 2–5, for increasing relaxation depths from 0 to 5 g/cm^2^, above a source energy of 50 keV. It is also shown that implementation of the calculated coefficients into the computation of the tissue weighting factors and the adult reference computational phantoms of ICRP Publication 103 does not significantly influence the effective dose conversion coefficients of the environment. Consequently, the coefficients shown in this paper could be applied for the evaluation of effective doses, as defined according to both recommendations of ICRP Publications 103 and 60.

## Introduction

Members of the public are exposed to various kinds of external photons in the environment, from both anthropogenic and natural radionuclides. For example, a small amount of anthropogenic radionuclides are constantly released from operating nuclear facilities, and in accidental situations, an even larger amount of radionuclides could be released. In terms of long-term exposure due to accidental releases, radionuclides deposited on the ground play an essential role, since they remain on and in ground and may result in an exposure of the public for a long time. It is well known, for example, that the area around the Chernobyl power plant is still contaminated, more than twenty-five years after the accident. Presently, the average annual external dose to residents of a rural settlement with substantial ^137^Cs deposition is estimated to be 0.9 mSv. Thus, for the critical group living in areas of high contamination and/or high transfer of radiocesium to foods, the dose value is considered to exceed the annual dose limit of 1 mSv set for the population (IAEA 2006). Also, in the Fukushima nuclear power plant accident that happened in 2011, wide regions were contaminated by radionuclides released into the atmosphere, and it is expected that in certain areas, the public could be exposed for a long time from the deposited radionuclides (Saito 2011).

Radionuclides in ground may show various distributions according to their origin and conditions: natural radionuclides such as ^40^K distribute rather uniformly in ground; in contrast, anthropogenic radionuclides deposited on ground distribute up to a depth of few tens of centimeters if the ground is plowed after deposition; further, in some cases, a peak in the radionuclides concentration is observed at a certain depth. However, in most cases, radionuclides deposited after an accident tend to show exponential distributions with depth in the ground (ICRU 1994).

Soon after deposition, radionuclides can be considered as a plane radioactive source on or in the ground. As time elapses, radionuclides deposited on the ground migrate into the soil and generally show a concentration distribution approximated by an exponential function with depth. Actually, after the Fukushima accident, many soil samples collected at different locations have been reported to show exponential distributions (MEXT 2012). Therefore, in order to evaluate doses to the public from long-term exposures due to contamination by accidents, basic data are necessary for dose evaluation of radionuclides exponentially distributed in ground.

Many studies have been carried out so far, in order to evaluate organ dose conversion coefficients for environmental exposures, including ground sources and air submersion (Poston and Snyder 1974; Dillman 1974; O’Brien and Sanna 1976; Koblinger and Nagy 1985; Jacob et al. 1986; DOE 1988; Saito et al. 1990, 1991, 1998, Jacob et al. 1990; Petoussi-Henss et al. 1991, 2008; Eckerman and Ryan 1993; Zankl et al. 1997; Petoussi-Henss and Saito 2009). While in earlier studies, the features of environmental gamma-ray fields were not described sufficiently (Poston and Snyder 1974; Dillman 1974; O’Brien and Sanna 1976; Koblinger and Nagy 1985; DOE 1988), several later studies provided dose coefficients considering the specific features of environmental gamma rays (i.e., energy and angular distributions) and using reliable Monte Carlo calculations (Jacob et al. 1986; Saito et al. 1990, 1991, 1998; Jacob et al. 1990; Petoussi-Henss 1991; Eckerman and Ryan 1993; Zankl et al. 1997; Petoussi-Henss et al. 2008; Petoussi-Henss and Saito 2009).

The radioactive sources considered in the studies mentioned above were mostly a submersion source in air (Poston and Snyder 1974; Dillman 1974; DOE 1988; Saito et al. 1990, 1991, 1998; Jacob et al. 1990; Petoussi-Henss et al. 1991, 2008; Eckerman and Ryan 1993; Zankl et al. 1997; Petoussi-Henss and Saito 2009), a plane source on or in ground (Koblinger and Nagy 1985; Jacob et al. 1986; DOE 1988; Saito et al. 1990, 1991, 1998; Jacob et al. 1990; Petoussi-Henss et al. 1991, 2008; Eckerman and Ryan 1993; Zankl et al. 1997; Petoussi-Henss and Saito 2009), and a volume ground source (O’Brien and Sanna 1976; Koblinger and Nagy 1985; Saito et al. 1990, 1991, 1998; Jacob et al. 1990; Petoussi-Henss et al. 1991, 2008 Eckerman and Ryan 1993; Zankl et al. 1997; Petoussi-Henss and Saito 2009) assuming mono-energetic photons or photons emitted by radionuclides. Extensive data have not been provided for exponential sources distributed in the ground. Furthermore, most of the studies have assumed exposure of adults, and only limited studies exist on exposures of children (Saito et al. 1990, 1991, 1998; Jacob et al. 1990; Petoussi-Henss 1991; Petoussi-Henss et al. 2008; Petoussi-Henss and Saito 2009).

The authors of the present paper have previously established methodologies for typical environmental sources such as (a) a submersion source in air, (b) a plane source in ground, and (c) a volume source in ground. This was done under consideration of the precise conditions of environmental photons, that is, energy spectrum and angular spectrum, and their change with height above the ground, as well as the transport of photons in the human phantoms (Saito et al. 1990, 1991, 1998; Jacob et al. 1990; Petoussi-Henss 1991; Zankl et al. 1997; Petoussi-Henss et al. 2008; Petoussi-Henss and Saito 2009). For sources (a) and (b), anthropogenic radionuclides were considered, while for source (c) natural radionuclides of the ^238^U series, the ^232^Th series, and ^40^K were considered.

In the present paper, effective dose conversion coefficients for radionuclides deposited exponentially in the ground are presented, for adults and a new-born baby. For this purpose, the coefficients were computed using dosimetric methodologies previously developed (Saito et al. 1990), the definition of effective dose as in the latest ICRP Recommendations (ICRP 2007), and updated nuclear data for dosimetric use (Endo 2003; ICRP 2008). Furthermore, the effect of body size was investigated by comparing the conversion coefficients for adults to those for infants. The difference of the respective effective doses computed according to ICRP Reports 103 (ICRP 2007) and 60 (ICRP 1991) definitions was investigated.

## Methods

Effective dose conversion coefficients for sources emitting mono-energetic photons can be estimated from the following equation:

1 $$ E(e,\alpha ) = E(e) \cdot K(e,\alpha ) $$

where *E* (*e,* α) is the conversion coefficient from unit photon emission per area to effective dose for a mono-energetic source exponentially distributed in ground at a relaxation depth of α (Sv per photon/m^2^); *e* is the photon energy (MeV); *E* (*e*) is the conversion coefficient from air kerma to effective dose for a plane source at a depth of 0.5 g/cm^2^ emitting mono-energetic photons (Sv/Gy); *K* (*e*,α) is the conversion coefficient from unit photon emission per area to air kerma for an exponentially distributed source at relaxation depth of α emitting mono-energetic photons with an energy of *e* (Gy per photon/m^2^).

Further, effective dose conversion coefficients for exponentially distributed radionuclides in the ground were evaluated by the following equation using basic data described below.

2 $$ E({\text{N}},\alpha ) = c\Upsigma y_{\text{Ni}} \cdot E(e_{\text{Ni}} ) \cdot K(e_{\text{Ni}} ,\alpha ) $$

where *E* (N,α) is the conversion coefficient from radioactivity per area to effective dose for radionuclide N exponentially distributed in ground at a relaxation depth of α (Sv/h per Bq/m^2^); *y*_Ni_ is the intensity of the i-th photon emitted from radionuclide N (per decay); *e*_Ni_ is the energy of the i-th photon emitted from nuclide N (MeV); *E* (*e*_Ni_) is the conversion coefficient from air kerma to effective dose for a plane source at a depth of 0.5 g/cm^2^ emitting mono-energetic photons with an energy of *e*_Ni_ (Sv/Gy); *K*(*e*_Ni_,α) is the conversion coefficient from unit photon emission per area to air kerma for an exponentially distributed source at a relaxation depth of α emitting monoe-nergetic photons with an energy of *e*_Ni_ (Gy per photon/m^2^); α is the relaxation depth of an exponentially distributed source (g/cm^2^); and *c* is a constant to adjust different time dimensions.

Equations 1 and 2 are based on the assumption that conversion coefficients from air kerma to effective dose for a plane source at a depth of 0.5 g/cm^2^ can represent those for exponential sources over a wide range of relaxation depths with negligible uncertainties; this will be discussed below. Relaxation depth refers to the depth where the radionuclide concentration reduces to 1/e relative to that at the ground surface, for an exponentially distributed source, *e* being Napier’s constant.

### Effective dose conversion coefficients for a plane source: *E* (*e*), *E* (*e*_Ni_)

A method for calculating organ doses considering the precise features of environmental photons has been previously established (Saito et al. 1990). In this method, the following three-step procedure was followed: (1) calculation of photon transport in the environment without the human body; (2) simulation of an imaginary cylindrical source around the phantom; (3) calculation of organ doses for photons emitted from the simulated cylindrical surface source (Saito et al. 1990).

This three-step procedure is advantageous for two reasons. First, the efficiency of environmental photon transport calculation is drastically enhanced without a human phantom. True infinity in the horizontal directions can be simulated without a human phantom, while finite cutoff in the horizontal directions must be set in transport calculation with a human phantom. This is because the situation that photons from a source having an infinite extent in the horizontal directions are detected by finite detectors is equivalent to the situation that photons from a finite source are detected by detectors having an infinite extent in the horizontal directions. Second, the same photon field can be repeatedly used once the field is obtained from transport calculations in the environment; in other words, a set of photon fields can be used for dose calculations for different phantoms and also for different postures by changing the size of the cylindrical source.

Environmental photon transport was previously simulated with the Monte Carlo code YURI (Saito and Moriuchi 1985) developed specially for environmental simulation. YURI, considering photo-electric absorption, Compton scattering and pair-production as photon interaction processes, has been verified by diverse experimental and computational data (Saito and Moriuchi 1985; Takada et al. 1985a, b; Saito et al. 1988; Saito 1991; Sakamoto and Saito 2003). Coherent scattering is not taken into account; however, neglecting coherent scattering would not affect the calculated photon fields, as proven by comparison with photon fields calculated with MCNP (Saito et al. 1990).

In the environmental simulation, an air-over-ground geometry where the interface between air and ground is considered to be an infinite plane was assumed (Saito et al. 1990). Air and soil were assumed to have constant densities of 1.2 × 10^−3^ g/cm^3^ and 1 g/cm^3^, respectively. If source depth is expressed in mass per area, soil density has been confirmed not to affect the calculated photon fields in air. For example, a source at 1 cm depth in soil having a density of 2 g/cm^3^ produces the same photon field in air as a source at 2 cm in soil having a density of 1 g/cm^3^, where the source depth expressed in mass per area is 2 g/cm^2^ for both cases. This fact allows a flexible description of soil density in the model calculations. Soil was assumed to consist of SiO_2_, Al_2_O_3_, Fe_2_O_3_ and H_2_O with weight fractions of 58.3, 16.7, 8.3 and 16.7 %, respectively.

From the resulting photon fields, a cylindrical surface source around the phantom was simulated so that it can reconstruct the original photon energy and angular distributions and their variation with height.

Petoussi-Henss and Saito (2009) and Petoussi-Henss et al. (2008) presented effective dose conversion coefficients based on the Reference Male and Reference Female phantoms (ICRP 2009) and the tissue weighting factors defined by ICRP 103 (ICRP 2007). In the present study, coefficients for babies were also calculated utilizing the voxel phantom Baby of an 8-week-old female baby (Veit et al. 1989). The Monte Carlo code EGSnrc (Kawrakow and Rogers 2003) was used for the transport of photons in the body. These data were utilized as *E* (*e*) and *E* (*e*_Ni_) in Eqs. (1) and (2), respectively.

The relationship of effective dose conversion coefficients between a plane source in the ground at a depth of 0.5 g/cm^2^ and exponential sources for different relaxation depths assuming mono-energetic photon emission has been already established (Saito et al. 1998). It was found that the conversion coefficients from air kerma to effective dose do not vary much with relaxation depth in ground. For relaxation depths up to 3 g/cm^2^, the conversion coefficients for exponential sources agree with those for plane sources at 0.5/cm^2^ within a few percent. The difference does not exceed 15 % even when a volume source is considered. This means that the maximum error inherent in the calculation of dose conversion coefficients is about 15 %. Further, if a typical relaxation depth of an exponential source up to 3 years after deposition is about 3 g/cm^2^, then the inherent errors due to folding of the Sv/Gy conversion coefficients would be within a few percent for most of typical exponential sources. Errors of this order would be sufficiently small from the viewpoint of environmental dose evaluation. Therefore, in the present study, air kerma to effective dose conversion coefficients for mono-energetic plane sources at 0.5 g/cm^2^ were applied for all sources characterized by an exponential distribution with different relaxation depths.

### Nuclear data: *y*_Ni_, *e*_Ni_

Endo and Yamaguchi compiled the nuclear data base DECDC (Nuclear Decay Data Files for Dosimetry Calculation) (Endo et al. 2003), implemented for preparing ICRP Publication 107 (ICRP 2009). The latter publication replaced ICRP Publication 38 (ICRP 1983) which has provided for many years the nuclear data for dosimetry in radiation protection and radiation medicine. DECDA was constructed by evaluating the nuclear data in the 1997 version of ENSDF (Evaluated Nuclear Structure Data File) (Tuli 2001), updated to include additional radionuclides. DECDC has been since utilized to calculate useful dosimetric data in various fields. For the present study, DECDC was utilized to obtain *y*_Ni_ and *e*_Ni_ in Eq. (2).

### Calculation of effective dose conversion coefficients for radionuclides

Effective dose conversion coefficients were calculated following the definition of the 2007 recommendations (ICRP 2007), using Eqs. (1) and (2). In order to enable users evaluation of effective doses for infants and estimation of the dose variation due to body size, the conversion coefficients were also calculated here for the Baby phantom. It should be noted that, however, effective dose is formally defined only for adults and is evaluated from the organ doses of the Reference Male and Reference Female phantoms.

Further, effective dose conversion coefficients were estimated according to the definition of ICRP Publication 60. In the present paper, *E*(*e*) was obtained by applying the tissue weighting factors defined in ICRP 60 to the same set of organ doses calculated using the Reference Male and Reference Female. The effective dose conversion coefficients according to ICRP Publication 60 obtained using stylized phantoms Adam and Eva (Kramer et al. 1982) were also cited for comparison.

In many countries including Japan and Germany, it takes a certain time to incorporate the ICRP recommendations of publication 103 into national law regulating radiation protection. Therefore, it is anticipated that the effective dose as defined in ICRP 60 would still be practically used for many years even from now. However, if the former and the more recent definition result in values of effective dose which are significantly different from each other, it would be controversial which data should be used. Thus, the new effective dose coefficients were compared with the old ones.

## Results and discussion

### Effective dose conversion coefficients for mono-energetic sources

**Fig. 1:**
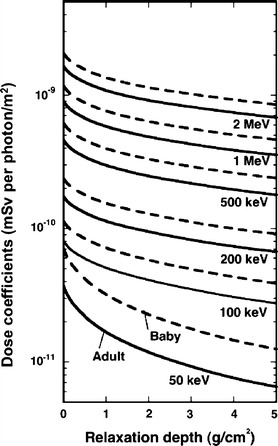
Effective dose conversion coefficients for exponentially distributed ground sources emitting mono-energetic photons

Figure 1 shows the effective dose conversion coefficients as a function of relaxation depth for mono-energetic exponential sources. The coefficients for Baby as well as those for adults are shown as effective dose per photon per area, for the energy range from 50 keV to 2 MeV. It can be seen that the coefficients are larger for higher energies. For example, the coefficient for adults at 2 MeV is 44 times larger than the coefficient at 50 keV for a surface plane source (α = 0), and 104 times larger for a relaxation depth of 5 g/cm^2^. This large difference is mainly due to the difference in air kerma per source intensity; furthermore, the variation in the self-shielding effect of the body as a function of photon energy enhances the difference.

The coefficients become smaller for larger relaxation depths because of the increasing shielding effect by soil, and this tendency is more pronounced at smaller source energies. For example, the coefficient at a relaxation depth of 5 g/cm^2^ is about 40 % of that at 0 g/cm^2^ for a 2 MeV source, while it is less than 20 % for a 50-keV source. The difference due to body size is also larger for smaller source energies because of the increasing self-shielding effect of the human body. For example, the coefficient for Baby is about 90 % larger than that for adults at 50 keV, while it is only 25 % larger at 2 MeV, independent of relaxation depth. Below 50 keV, the difference increases further. These characteristics will be obviously reflected by coefficients for radionuclides, depending on the emitted energy spectra.

### Effective dose conversion coefficients for radionuclides

**Fig. 2:**
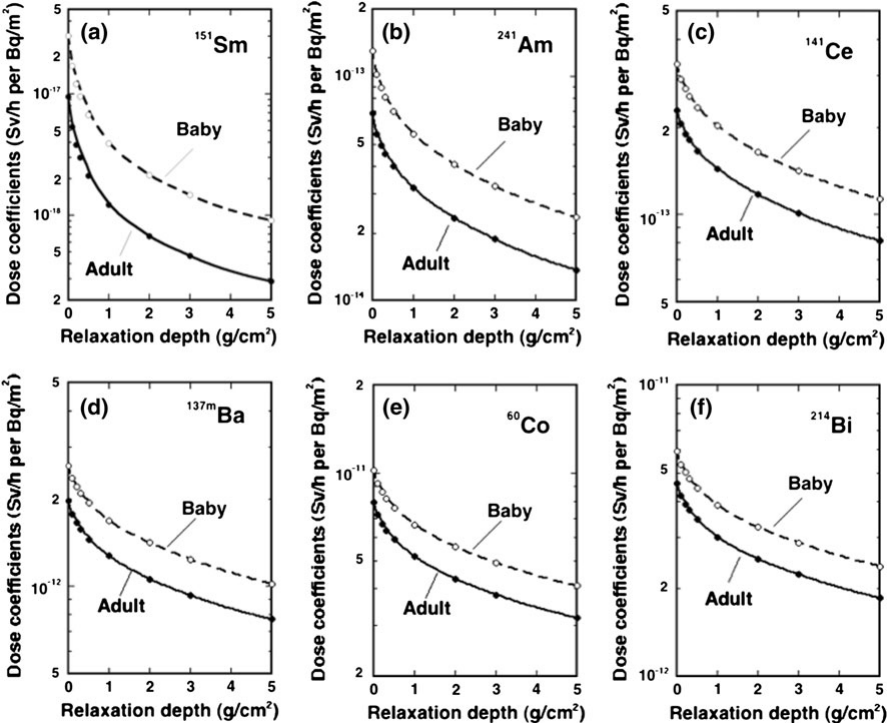
Effective dose conversion coefficients for several selected radionuclides exponentially distributed in ground

The effective dose conversion coefficients were evaluated for 185 nuclides that are potentially important as ground sources. In the present paper, effective dose conversion coefficients for some selected radionuclides are tabulated in Tables 1 and 2 for adults and for the Baby, respectively. Furthermore, the coefficients for ^151^Sm, ^241^Am, ^141^Ce, ^137m^Ba, ^60^Co, and ^214^Bi are illustrated as a function of relaxation depth in Fig. 2. Except ^214^Bi, these are all anthropogenic radionuclides that could be eventually released as a result of accidental exposures.

**Table 1:** Effective dose conversion coefficients for adults due to photons from radionuclides exponentially distributed in the ground

Nuclide	Dose conversion coefficients (Sv/h per Bq/m^2^)													
	Relaxation depth (g/cm^2^)													
	0	0.1	0.2	0.3	0.5	1.0	2.0	3.0	5.0	10	20	30	50	100
^7^Be	1.66E−13	1.50E−13	1.40E−13	1.33E−13	1.23E−13	1.07E−13	8.94E−14	7.87E−14	6.45E−14	4.62E−14	3.03E−14	2.26E−14	1.51E−14	8.24E−15
^22^Na	7.08E−12	6.41E−12	6.00E−12	5.69E−12	5.27E−12	4.61E−12	3.86E−12	3.40E−12	2.81E−12	2.04E−12	1.36E−12	1.03E−12	6.97E−13	3.87E−13
^46^Sc	6.47E−12	5.86E−12	5.48E−12	5.21E−12	4.82E−12	4.21E−12	3.53E−12	3.11E−12	2.58E−12	1.88E−12	1.26E−12	9.56E−13	6.48E−13	3.60E−13
^51^Cr	1.03E−13	9.35E−14	8.74E−14	8.30E−14	7.70E−14	6.71E−14	5.59E−14	4.91E−14	4.00E−14	2.85E−14	1.83E−14	1.36E−14	9.00E−15	4.89E−15
^54^Ma	2.72E−12	2.46E−12	2.30E−12	2.19E−12	2.02E−12	1.77E−12	1.48E−12	1.30E−12	1.08E−12	7.82E−13	5.18E−13	3.92E−13	2.66E−13	1.47E−13
^59^Fe	3.79E−12	3.43E−12	3.21E−12	3.04E−12	2.82E−12	2.47E−12	2.07E−12	1.83E−12	1.52E−12	1.11E−12	7.48E−13	5.71E−13	3.88E−13	2.16E−13
^57^Co	3.59E−13	3.25E−13	3.04E−13	2.89E−13	2.66E−13	2.31E−13	1.89E−13	1.63E−13	1.30E−13	8.83E−14	5.43E−14	3.92E−14	2.53E−14	1.34E−14
^58^Co	3.18E−12	2.88E−12	2.70E−12	2.56E−12	2.37E−12	2.06E−12	1.73E−12	1.52E−12	1.26E−12	9.10E−13	6.02E−13	4.54E−13	3.07E−13	1.69E−13
^60^Co	7.96E−12	7.21E−12	6.75E−12	6.40E−12	5.94E−12	5.20E−12	4.37E−12	3.85E−12	3.20E−12	2.35E−12	1.58E−12	1.21E−12	8.23E−13	4.60E−13
^65^Zn	1.85E−12	1.68E−12	1.57E−12	1.49E−12	1.38E−12	1.21E−12	1.01E−12	8.93E−13	7.41E−13	5.40E−13	3.63E−13	2.77E−13	1.88E−13	1.04E−13
^86^Rb	2.98E−13	2.70E−13	2.52E−13	2.40E−13	2.22E−13	1.94E−13	1.63E−13	1.43E−13	1.19E−13	8.67E−14	5.83E−14	4.44E−14	3.01E−14	1.67E−14
^85^Sr	1.63E−12	1.47E−12	1.38E−12	1.31E−12	1.21E−12	1.05E−12	8.77E−13	7.72E−13	6.34E−13	4.54E−13	2.98E−13	2.23E−13	1.49E−13	8.15E−14
^89^Sr	2.81E−16	2.55E−16	2.38E−16	2.26E−16	2.09E−16	1.82E−16	1.53E−16	1.35E−16	1.12E−16	8.11E−17	5.40E−17	4.09E−17	2.77E−17	1.53E−17
^88^Y	8.27E−12	7.49E−12	7.03E−12	6.69E−12	6.20E−12	5.42E−12	4.56E−12	4.05E−12	3.37E−12	2.48E−12	1.68E−12	1.29E−12	8.83E−13	4.97E−13
^90^Y	1.02E−18	5.24E−19	3.62E−19	2.85E−19	2.07E−19	1.35E−19	8.92E−20	7.11E−20	5.33E−20	3.58E−20	2.31E−20	1.74E−20	1.18E−20	6.62E−21
^91^Y	1.15E−14	1.04E−14	9.76E−15	9.26E−15	8.59E−15	7.52E−15	6.31E−15	5.57E−15	4.63E−15	3.38E−15	2.28E−15	1.74E−15	1.18E−15	6.60E−16
^89^Zr	3.77E−12	3.41E−12	3.19E−12	3.03E−12	2.80E−12	2.44E−12	2.05E−12	1.80E−12	1.49E−12	1.08E−12	7.17E−13	5.42E−13	3.67E−13	2.03E−13
^95^Zr	2.40E−12	2.18E−12	2.04E−12	1.93E−12	1.79E−12	1.56E−12	1.31E−12	1.15E−12	9.50E−13	6.87E−13	4.53E−13	3.42E−13	2.31E−13	1.27E−13
^94^Nb	5.09E−12	4.61E−12	4.31E−12	4.10E−12	3.79E−12	3.30E−12	2.77E−12	2.44E−12	2.02E−12	1.46E−12	9.66E−13	7.29E−13	4.93E−13	2.72E−13
^95m^Nb	2.08E−13	1.86E−13	1.74E−13	1.65E−13	1.52E−13	1.32E−13	1.10E−13	9.61E−14	7.84E−14	5.51E−14	3.50E−14	2.58E−14	1.70E−14	9.17E−15
^95^Nb	2.50E−12	2.27E−12	2.12E−12	2.02E−12	1.86E−12	1.63E−12	1.36E−12	1.20E−12	9.92E−13	7.18E−13	4.74E−13	3.57E−13	2.42E−13	1.33E−13
^99^Mo	4.81E−13	4.36E−13	4.08E−13	3.87E−13	3.58E−13	3.12E−13	2.61E−13	2.29E−13	1.89E−13	1.36E−13	8.92E−14	6.70E−14	4.51E−14	2.48E−14
^103^Ru	1.64E−12	1.48E−12	1.38E−12	1.32E−12	1.21E−12	1.06E−12	8.83E−13	7.77E−13	6.38E−13	4.57E−13	3.00E−13	2.24E−13	1.50E−13	8.20E−14
^106^Rh	6.76E−13	6.11E−13	5.72E−13	5.43E−13	5.02E−13	4.38E−13	3.66E−13	3.22E−13	2.65E−13	1.91E−13	1.26E−13	9.46E−14	6.36E−14	3.50E−14
^108m^Ag	5.31E−12	4.80E−12	4.49E−12	4.26E−12	3.93E−12	3.43E−12	2.87E−12	2.52E−12	2.08E−12	1.50E−12	9.81E−13	7.36E−13	4.94E−13	2.72E−13
^110m^Ag	8.81E−12	7.98E−12	7.47E−12	7.09E−12	6.56E−12	5.73E−12	4.81E−12	4.23E−12	3.51E−12	2.55E−12	1.69E−12	1.28E−12	8.69E−13	4.82E−13
^111^Ag	8.61E−14	7.79E−14	7.28E−14	6.92E−14	6.41E−14	5.59E−14	4.65E−14	4.08E−14	3.33E−14	2.37E−14	1.53E−14	1.13E−14	7.51E−15	4.08E−15
^109^Cd	4.38E−14	2.83E−14	2.22E−14	1.88E−14	1.49E−14	1.05E−14	7.23E−15	5.70E−15	4.11E−15	2.50E−15	1.43E−15	1.00E−15	6.30E−16	3.27E−16
^124^Sb	5.84E−12	5.29E−12	4.96E−12	4.72E−12	4.37E−12	3.82E−12	3.21E−12	2.84E−12	2.35E−12	1.72E−12	1.16E−12	8.79E−13	5.99E−13	3.35E−13
^125^Sb	1.42E−12	1.27E−12	1.19E−12	1.13E−12	1.04E−12	9.04E−13	7.53E−13	6.61E−13	5.43E−13	3.89E−13	2.54E−13	1.90E−13	1.27E−13	6.96E−14
^127^Sb	2.28E−12	2.06E−12	1.93E−12	1.83E−12	1.69E−12	1.47E−12	1.23E−12	1.08E−12	8.93E−13	6.43E−13	4.23E−13	3.17E−13	2.13E−13	1.17E−13
^123m^Te	4.38E−13	3.90E−13	3.63E−13	3.43E−13	3.15E−13	2.73E−13	2.25E−13	1.95E−13	1.57E−13	1.08E−13	6.74E−14	4.91E−14	3.20E−14	1.70E−14
^127m^Te	2.24E−14	1.50E−14	1.18E−14	9.95E−15	7.71E−15	5.08E−15	3.13E−15	2.29E−15	1.52E−15	8.40E−16	4.52E−16	3.10E−16	1.92E−16	9.84E−17
^127^Te	1.60E−14	1.44E−14	1.35E−14	1.28E−14	1.18E−14	1.03E−14	8.58E−15	7.55E−15	6.16E−15	4.40E−15	2.87E−15	2.13E−15	1.42E−15	7.72E−16
^129m^Te	1.13E−13	9.81E−14	9.03E−14	8.48E−14	7.73E−14	6.62E−14	5.47E−14	4.78E−14	3.92E−14	2.82E−14	1.85E−14	1.39E−14	9.36E−15	5.15E−15
^129^Te	1.98E−13	1.77E−13	1.64E−13	1.55E−13	1.43E−13	1.24E−13	1.03E−13	9.05E−14	7.42E−14	5.31E−14	3.49E−14	2.61E−14	1.74E−14	9.55E−15
^132^Te	7.11E−13	6.34E−13	5.90E−13	5.58E−13	5.12E−13	4.41E−13	3.65E−13	3.17E−13	2.57E−13	1.79E−13	1.13E−13	8.35E−14	5.48E−14	2.96E−14
^129^I	5.38E−14	3.76E−14	3.01E−14	2.56E−14	2.00E−14	1.33E−14	8.17E−15	5.92E−15	3.83E−15	2.04E−15	1.06E−15	7.11E−16	4.32E−16	2.18E−16
^131^I	1.25E−12	1.13E−12	1.06E−12	1.00E−12	9.29E−13	8.10E−13	6.74E−13	5.93E−13	4.85E−13	3.46E−13	2.25E−13	1.67E−13	1.11E−13	6.06E−14
^132^I	7.37E−12	6.67E−12	6.24E−12	5.93E−12	5.48E−12	4.79E−12	4.01E−12	3.53E−12	2.92E−12	2.12E−12	1.40E−12	1.06E−12	7.16E−13	3.96E−13
^134^Cs	5.10E−12	4.62E−12	4.32E−12	4.10E−12	3.79E−12	3.31E−12	2.77E−12	2.44E−12	2.01E−12	1.45E−12	9.59E−13	7.22E−13	4.87E−13	2.69E−13
^136^Cs	6.86E−12	6.22E−12	5.81E−12	5.52E−12	5.10E−12	4.46E−12	3.73E−12	3.29E−12	2.72E−12	1.97E−12	1.31E−12	9.94E−13	6.72E−13	3.72E−13
^133^Ba	1.26E−12	1.12E−12	1.04E−12	9.81E−13	9.02E−13	7.78E−13	6.40E−13	5.59E−13	4.53E−13	3.20E−13	2.05E−13	1.52E−13	1.00E−13	5.44E−14
^137m^Ba	1.96E−12	1.78E−12	1.66E−12	1.58E−12	1.46E−12	1.27E−12	1.07E−12	9.35E−13	7.73E−13	5.57E−13	3.66E−13	2.75E−13	1.85E−13	1.02E−13
^140^Ba	5.90E−13	5.31E−13	4.96E−13	4.70E−13	4.34E−13	3.78E−13	3.14E−13	2.76E−13	2.26E−13	1.62E−13	1.06E−13	7.88E−14	5.26E−14	2.88E−14
^140^La	7.21E−12	6.53E−12	6.13E−12	5.82E−12	5.40E−12	4.72E−12	3.97E−12	3.51E−12	2.92E−12	2.14E−12	1.44E−12	1.10E−12	7.52E−13	4.21E−13
^141^La	8.39E−14	7.60E−14	7.13E−14	6.76E−14	6.28E−14	5.50E−14	4.63E−14	4.09E−14	3.41E−14	2.50E−14	1.70E−14	1.30E−14	8.87E−15	4.98E−15
^141^Ce	2.28E−13	2.05E−13	1.90E−13	1.81E−13	1.65E−13	1.44E−13	1.17E−13	1.01E−13	8.12E−14	5.55E−14	3.44E−14	2.50E−14	1.62E−14	8.61E−15
^144^Ce	5.60E−14	4.95E−14	4.56E−14	4.29E−14	3.90E−14	3.34E−14	2.69E−14	2.30E−14	1.82E−14	1.23E−14	7.52E−15	5.42E−15	3.50E−15	1.85E−15
^144^Pr	8.83E−14	8.00E−14	7.51E−14	7.14E−14	6.62E−14	5.79E−14	4.87E−14	4.33E−14	3.60E−14	2.65E−14	1.79E−14	1.37E−14	9.41E−15	5.29E−15
^147^Nd	4.38E−13	3.89E−13	3.60E−13	3.40E−13	3.11E−13	2.67E−13	2.19E−13	1.90E−13	1.53E−13	1.07E−13	6.89E−14	5.11E−14	3.39E−14	1.84E−14
^151^Sm	9.49E−18	5.34E−18	3.80E−18	2.99E−18	2.11E−18	1.22E−18	6.70E−19	4.62E−19	2.85E−19	1.45E−19	7.37E−20	4.92E−20	2.96E−20	1.48E−20
^152^Eu	3.73E−12	3.37E−12	3.15E−12	2.98E−12	2.76E−12	2.41E−12	2.01E−12	1.77E−12	1.46E−12	1.06E−12	7.07E−13	5.36E−13	3.62E−13	2.01E−13
^154^Eu	3.60E−12	3.25E−12	3.04E−12	2.89E−12	2.67E−12	2.33E−12	1.95E−12	1.72E−12	1.42E−12	1.03E−12	6.88E−13	5.22E−13	3.53E−13	1.96E−13
^155^Eu	1.72E−13	1.52E−13	1.40E−13	1.31E−13	1.19E−13	1.00E−13	7.92E−14	6.67E−14	5.14E−14	3.32E−14	1.98E−14	1.40E−14	8.93E−15	4.68E−15
^156^Eu	3.80E−12	3.44E−12	3.22E−12	3.06E−12	2.84E−12	2.48E−12	2.09E−12	1.85E−12	1.53E−12	1.13E−12	7.61E−13	5.82E−13	3.98E−13	2.23E−13
^160^Tb	3.61E−12	3.27E−12	3.06E−12	2.90E−12	2.69E−12	2.34E−12	1.96E−12	1.73E−12	1.43E−12	1.04E−12	6.90E−13	5.23E−13	3.54E−13	1.96E−13
^169^Yb	9.50E−13	8.39E−13	7.74E−13	7.28E−13	6.60E−13	5.57E−13	4.44E−13	3.77E−13	2.96E−13	1.98E−13	1.21E−13	8.77E−14	5.68E−14	3.02E−14
^181^Hf	1.72E−12	1.55E−12	1.45E−12	1.38E−12	1.27E−12	1.11E−12	9.18E−13	8.05E−13	6.57E−13	4.66E−13	3.03E−13	2.25E−13	1.49E−13	8.14E−14
^182^Ta	4.08E−12	3.69E−12	3.45E−12	3.27E−12	3.03E−12	2.64E−12	2.21E−12	1.95E−12	1.61E−12	1.17E−12	7.82E−13	5.95E−13	4.02E−13	2.24E−13
^192^Ir	2.67E−12	2.41E−12	2.26E−12	2.14E−12	1.98E−12	1.73E−12	1.44E−12	1.27E−12	1.03E−12	7.38E−13	4.78E−13	3.55E−13	2.36E−13	1.29E−13
^203^Hg	7.59E−13	6.89E−13	6.45E−13	6.12E−13	5.67E−13	4.93E−13	4.10E−13	3.59E−13	2.92E−13	2.07E−13	1.32E−13	9.74E−14	6.43E−14	3.48E−14
^214^Pb	8.11E−13	7.33E−13	6.85E−13	6.50E−13	6.01E−13	5.23E−13	4.34E−13	3.80E−13	3.10E−13	2.20E−13	1.41E−13	1.05E−13	6.92E−14	3.76E−14
^214^Bi	4.62E−12	4.19E−12	3.92E−12	3.73E−12	3.46E−12	3.02E−12	2.54E−12	2.25E−12	1.87E−12	1.37E−12	9.24E−13	7.05E−13	4.81E−13	2.69E−13
^222^Rn	1.29E−15	1.16E−15	1.09E−15	1.03E−15	9.51E−16	8.30E−16	6.92E−16	6.09E−16	5.00E−16	3.58E−16	2.35E−16	1.76E−16	1.17E−16	6.43E−17
^239^Np	5.35E−13	4.81E−13	4.49E−13	4.26E−13	3.92E−13	3.39E−13	2.79E−13	2.42E−13	1.94E−13	1.34E−13	8.36E−14	6.10E−14	3.98E−14	2.13E−14
^238^Pu	1.54E−15	7.87E−16	5.33E−16	4.09E−16	2.84E−16	1.67E−16	9.59E−17	6.87E−17	4.47E−17	2.45E−17	1.31E−17	8.97E−18	5.52E−18	2.82E−18
^239^Pu	8.40E−16	4.98E−16	3.78E−16	3.17E−16	2.52E−16	1.83E−16	1.31E−16	1.07E−16	8.08E−17	5.27E−17	3.21E−17	2.32E−17	1.50E−17	7.98E−18
^240^Pu	1.47E−15	7.52E−16	5.11E−16	3.94E−16	2.76E−16	1.63E−16	9.49E−17	6.83E−17	4.48E−17	2.47E−17	1.33E−17	9.14E−18	5.64E−18	2.89E−18
^241^Pu	4.57E−18	4.06E−18	3.76E−18	3.56E−18	3.26E−18	2.80E−18	2.28E−18	1.95E−18	1.54E−18	1.02E−18	6.21E−19	4.45E−19	2.86E−19	1.51E−19
^241^Am	6.87E−14	5.54E−14	4.93E−14	4.53E−14	3.98E−14	3.17E−14	2.34E−14	1.88E−14	1.37E−14	8.20E−15	4.59E−15	3.19E−15	1.98E−15	1.02E−15
^242^Cm	1.71E−15	8.89E−16	6.06E−16	4.67E−16	3.25E−16	1.89E−16	1.07E−16	7.58E−17	4.88E−17	2.65E−17	1.42E−17	9.70E−18	5.99E−18	3.07E−18

**Table 2:** Effective dose conversion coefficients for Baby due to photons from radionuclides exponentially distributed in the ground

Nuclide	Dose conversion coefficients (Sv/h per Bq/m^2^)													
	Relaxation depth (g/cm^2^)													
	0	0.1	0.2	0.3	0.5	1.0	2.0	3.0	5.0	10	20	30	50	100
^7^Be	2.23E−13	2.01E−13	1.88E−13	1.79E−13	1.65E−13	1.44E−13	1.20E−13	1.06E−13	8.66E−14	6.20E−14	4.06E−14	3.04E−14	2.02E−14	1.11E−14
^22^Na	9.25E−12	8.37E−12	7.83E−12	7.43E−12	6.88E−12	6.01E−12	5.04E−12	4.44E−12	3.67E−12	2.67E−12	1.78E−12	1.35E−12	9.09E−13	5.04E−13
^46^Sc	8.42E−12	7.63E−12	7.13E−12	6.77E−12	6.27E−12	5.47E−12	4.59E−12	4.05E−12	3.36E−12	2.44E−12	1.64E−12	1.24E−12	8.43E−13	4.68E−13
^51^Cr	1.41E−13	1.27E−13	1.19E−13	1.13E−13	1.05E−13	9.14E−14	7.61E−14	6.68E−14	5.45E−14	3.89E−14	2.50E−14	1.85E−14	1.23E−14	6.66E−15
^54^Ma	3.55E−12	3.22E−12	3.01E−12	2.87E−12	2.65E−12	2.31E−12	1.94E−12	1.70E−12	1.41E−12	1.02E−12	6.78E−13	5.12E−13	3.47E−13	1.92E−13
^59^Fe	4.89E−12	4.43E−12	4.14E−12	3.93E−12	3.65E−12	3.19E−12	2.68E−12	2.36E−12	1.96E−12	1.43E−12	9.66E−13	7.37E−13	5.00E−13	2.79E−13
^57^Co	4.97E−13	4.50E−13	4.20E−13	3.99E−13	3.67E−13	3.19E−13	2.61E−13	2.25E−13	1.80E−13	1.22E−13	7.50E−14	5.41E−14	3.50E−14	1.85E−14
^58^Co	4.17E−12	3.78E−12	3.53E−12	3.36E−12	3.10E−12	2.71E−12	2.27E−12	2.00E−12	1.65E−12	1.19E−12	7.89E−13	5.95E−13	4.02E−13	2.22E−13
^60^Co	1.02E−11	9.26E−12	8.67E−12	8.22E−12	7.63E−12	6.68E−12	5.61E−12	4.95E−12	4.12E−12	3.01E−12	2.04E−12	1.56E−12	1.06E−12	5.91E−13
^65^Zn	2.41E−12	2.18E−12	2.04E−12	1.93E−12	1.79E−12	1.57E−12	1.31E−12	1.16E−12	9.61E−13	7.01E−13	4.72E−13	3.59E−13	2.44E−13	1.35E−13
^86^Rb	3.88E−13	3.51E−13	3.28E−13	3.12E−13	2.89E−13	2.52E−13	2.12E−13	1.87E−13	1.55E−13	1.13E−13	7.58E−14	5.77E−14	3.91E−14	2.17E−14
^85^Sr	2.18E−12	1.97E−12	1.84E−12	1.75E−12	1.61E−12	1.40E−12	1.17E−12	1.03E−12	8.47E−13	6.07E−13	3.98E−13	2.97E−13	1.99E−13	1.09E−13
^89^Sr	3.67E−16	3.33E−16	3.11E−16	2.96E−16	2.73E−16	2.38E−16	2.00E−16	1.76E−16	1.46E−16	1.06E−16	7.05E−17	5.34E−17	3.63E−17	2.00E−17
^88^Y	1.05E−11	9.54E−12	8.95E−12	8.52E−12	7.89E−12	6.90E−12	5.80E−12	5.16E−12	4.29E−12	3.16E−12	2.14E−12	1.64E−12	1.12E−12	6.31E−13
^90^Y	2.99E−18	1.47E−18	9.79E−19	7.46E−19	5.17E−19	3.07E−19	1.84E−19	1.38E−19	9.65E−20	5.98E−20	3.64E−20	2.68E−20	1.78E−20	9.77E−21
^91^Y	1.49E−14	1.35E−14	1.26E−14	1.19E−14	1.11E−14	9.69E−15	8.14E−15	7.18E−15	5.96E−15	4.36E−15	2.94E−15	2.25E−15	1.53E−15	8.51E−16
^89^Zr	4.96E−12	4.48E−12	4.19E−12	3.98E−12	3.68E−12	3.21E−12	2.69E−12	2.37E−12	1.96E−12	1.42E−12	9.41E−13	7.11E−13	4.81E−13	2.66E−13
^95^Zr	3.14E−12	2.85E−12	2.66E−12	2.53E−12	2.34E−12	2.04E−12	1.71E−12	1.50E−12	1.24E−12	8.99E−13	5.93E−13	4.47E−13	3.02E−13	1.67E−13
^94^Nb	6.66E−12	6.03E−12	5.64E−12	5.37E−12	4.95E−12	4.32E−12	3.62E−12	3.19E−12	2.64E−12	1.91E−12	1.26E−12	9.54E−13	6.46E−13	3.56E−13
^95m^Nb	2.94E−13	2.59E−13	2.41E−13	2.28E−13	2.09E−13	1.81E−13	1.51E−13	1.31E−13	1.07E−13	7.51E−14	4.77E−14	3.52E−14	2.31E−14	1.25E−14
^95^Nb	3.28E−12	2.97E−12	2.78E−12	2.64E−12	2.44E−12	2.13E−12	1.78E−12	1.57E−12	1.30E−12	9.39E−13	6.20E−13	4.67E−13	3.16E−13	1.74E−13
^99^Mo	6.36E−13	5.75E−13	5.38E−13	5.11E−13	4.71E−13	4.11E−13	3.44E−13	3.02E−13	2.49E−13	1.79E−13	1.17E−13	8.81E−14	5.93E−14	3.26E−14
^103^Ru	2.19E−12	1.98E−12	1.85E−12	1.76E−12	1.62E−12	1.42E−12	1.18E−12	1.04E−12	8.54E−13	6.12E−13	4.01E−13	3.00E−13	2.00E−13	1.10E−13
^106^Rh	8.94E−13	8.08E−13	7.56E−13	7.18E−13	6.64E−13	5.79E−13	4.84E−13	4.26E−13	3.51E−13	2.53E−13	1.67E−13	1.25E−13	8.40E−14	4.63E−14
^108m^Ag	7.06E−12	6.37E−12	5.95E−12	5.65E−12	5.21E−12	4.54E−12	3.80E−12	3.34E−12	2.75E−12	1.98E−12	1.30E−12	9.73E−13	6.54E−13	3.59E−13
^110m^Ag	1.15E−11	1.04E−11	9.72E−12	9.24E−12	8.54E−12	7.46E−12	6.26E−12	5.51E−12	4.56E−12	3.32E−12	2.20E−12	1.67E−12	1.13E−12	6.27E−13
^111^Ag	1.17E−13	1.06E−13	9.91E−14	9.41E−14	8.72E−14	7.61E−14	6.32E−14	5.56E−14	4.53E−14	3.23E−14	2.08E−14	1.54E−14	1.02E−14	5.55E−15
^109^Cd	1.23E−13	7.55E−14	5.72E−14	4.71E−14	3.59E−14	2.37E−14	1.52E−14	1.15E−14	7.96E−15	4.65E−15	2.60E−15	1.80E−15	1.12E−15	5.79E−16
^124^Sb	7.52E−12	6.81E−12	6.38E−12	6.07E−12	5.62E−12	4.91E−12	4.13E−12	3.65E−12	3.03E−12	2.21E−12	1.49E−12	1.13E−12	7.69E−13	4.30E−13
^125^Sb	1.94E−12	1.73E−12	1.61E−12	1.52E−12	1.40E−12	1.22E−12	1.01E−12	8.86E−13	7.26E−13	5.20E−13	3.40E−13	2.54E−13	1.70E−13	9.30E−14
^127^Sb	3.01E−12	2.72E−12	2.55E−12	2.42E−12	2.24E−12	1.95E−12	1.63E−12	1.43E−12	1.18E−12	8.50E−13	5.58E−13	4.19E−13	2.82E−13	1.55E−13
^123m^Te	6.43E−13	5.62E−13	5.18E−13	4.88E−13	4.44E−13	3.82E−13	3.12E−13	2.70E−13	2.17E−13	1.49E−13	9.26E−14	6.75E−14	4.39E−14	2.34E−14
^127m^Te	6.41E−14	4.26E−14	3.33E−14	2.79E−14	2.14E−14	1.39E−14	8.37E−15	6.06E−15	3.93E−15	2.12E−15	1.12E−15	7.60E−16	4.66E−16	2.37E−16
^127^Te	2.18E−14	1.96E−14	1.83E−14	1.74E−14	1.61E−14	1.40E−14	1.16E−14	1.02E−14	8.35E−15	5.96E−15	3.88E−15	2.89E−15	1.92E−15	1.05E−15
^129m^Te	1.73E−13	1.45E−13	1.31E−13	1.22E−13	1.09E−13	9.19E−14	7.47E−14	6.48E−14	5.28E−14	3.76E−14	2.46E−14	1.84E−14	1.24E−14	6.83E−15
^129^Te	2.80E−13	2.46E−13	2.27E−13	2.14E−13	1.96E−13	1.69E−13	1.40E−13	1.22E−13	1.00E−13	7.15E−14	4.68E−14	3.50E−14	2.34E−14	1.28E−14
^132^Te	1.05E−12	9.17E−13	8.47E−13	7.97E−13	7.27E−13	6.20E−13	5.09E−13	4.40E−13	3.56E−13	2.48E−13	1.56E−13	1.15E−13	7.53E−14	4.06E−14
^129^I	1.47E−13	1.03E−13	8.20E−14	6.96E−14	5.44E−14	3.61E−14	2.20E−14	1.60E−14	1.03E−14	5.48E−15	2.84E−15	1.91E−15	1.16E−15	5.83E−16
^131^I	1.70E−12	1.53E−12	1.43E−12	1.36E−12	1.26E−12	1.10E−12	9.12E−13	8.02E−13	6.55E−13	4.68E−13	3.04E−13	2.26E−13	1.50E−13	8.18E−14
^132^I	9.63E−12	8.72E−12	8.16E−12	7.75E−12	7.17E−12	6.25E−12	5.24E−12	4.62E−12	3.82E−12	2.77E−12	1.83E−12	1.38E−12	9.35E−13	5.17E−13
^134^Cs	6.70E−12	6.06E−12	5.67E−12	5.39E−12	4.98E−12	4.34E−12	3.64E−12	3.20E−12	2.64E−12	1.91E−12	1.26E−12	9.48E−13	6.40E−13	3.53E−13
^136^Cs	8.99E−12	8.15E−12	7.61E−12	7.23E−12	6.69E−12	5.84E−12	4.89E−12	4.30E−12	3.56E−12	2.58E−12	1.72E−12	1.30E−12	8.79E−13	4.86E−13
^133^Ba	1.84E−12	1.62E−12	1.49E−12	1.40E−12	1.28E−12	1.09E−12	8.94E−13	7.78E−13	6.27E−13	4.42E−13	2.83E−13	2.09E−13	1.38E−13	7.47E−14
^137m^Ba	2.59E−12	2.34E−12	2.19E−12	2.08E−12	1.92E−12	1.67E−12	1.40E−12	1.23E−12	1.02E−12	7.32E−13	4.81E−13	3.61E−13	2.43E−13	1.34E−13
^140^Ba	8.06E−13	7.21E−13	6.72E−13	6.36E−13	5.86E−13	5.09E−13	4.23E−13	3.71E−13	3.04E−13	2.17E−13	1.41E−13	1.06E−13	7.04E−14	3.85E−14
^140^La	9.25E−12	8.38E−12	7.85E−12	7.46E−12	6.92E−12	6.05E−12	5.09E−12	4.50E−12	3.74E−12	2.74E−12	1.85E−12	1.41E−12	9.62E−13	5.39E−13
^141^La	1.07E−13	9.68E−14	9.08E−14	8.61E−14	8.00E−14	7.00E−14	5.89E−14	5.21E−14	4.34E−14	3.19E−14	2.16E−14	1.66E−14	1.13E−14	6.34E−15
^141^Ce	3.30E−13	2.93E−13	2.71E−13	2.56E−13	2.34E−13	2.02E−13	1.64E−13	1.41E−13	1.13E−13	7.68E−14	4.75E−14	3.45E−14	2.24E−14	1.19E−14
^144^Ce	8.61E−14	7.49E−14	6.84E−14	6.40E−14	5.77E−14	4.88E−14	3.88E−14	3.30E−14	2.59E−14	1.74E−14	1.06E−14	7.64E−15	4.93E−15	2.60E−15
^144^Pr	1.12E−13	1.02E−13	9.54E−14	9.08E−14	8.41E−14	7.36E−14	6.19E−14	5.49E−14	4.57E−14	3.37E−14	2.28E−14	1.74E−14	1.19E−14	6.71E−15
^147^Nd	6.38E−13	5.62E−13	5.17E−13	4.85E−13	4.42E−13	3.76E−13	3.06E−13	2.64E−13	2.12E−13	1.48E−13	9.44E−14	6.98E−14	4.62E−14	2.51E−14
^151^Sm	3.02E−17	1.70E−17	1.21E−17	9.52E−18	6.72E−18	3.90E−18	2.14E−18	1.47E−18	9.07E−19	4.63E−19	2.35E−19	1.57E−19	9.45E−20	4.73E−20
^152^Eu	4.94E−12	4.45E−12	4.15E−12	3.93E−12	3.63E−12	3.16E−12	2.64E−12	2.32E−12	1.91E−12	1.39E−12	9.23E−13	6.99E−13	4.73E−13	2.62E−13
^154^Eu	4.71E−12	4.26E−12	3.98E−12	3.77E−12	3.49E−12	3.04E−12	2.55E−12	2.24E−12	1.85E−12	1.34E−12	8.95E−13	6.79E−13	4.59E−13	2.55E−13
^155^Eu	2.66E−13	2.33E−13	2.14E−13	2.00E−13	1.81E−13	1.51E−13	1.19E−13	9.94E−14	7.62E−14	4.91E−14	2.91E−14	2.06E−14	1.31E−14	6.86E−15
^156^Eu	4.88E−12	4.41E−12	4.13E−12	3.93E−12	3.64E−12	3.18E−12	2.67E−12	2.37E−12	1.96E−12	1.44E−12	9.74E−13	7.44E−13	5.08E−13	2.85E−13
^160^Tb	4.74E−12	4.29E−12	4.01E−12	3.81E−12	3.52E−12	3.07E−12	2.57E−12	2.26E−12	1.87E−12	1.36E−12	9.02E−13	6.84E−13	4.62E−13	2.56E−13
^169^Yb	1.46E−12	1.28E−12	1.18E−12	1.11E−12	1.00E−12	8.39E−13	6.63E−13	5.59E−13	4.36E−13	2.89E−13	1.76E−13	1.27E−13	8.19E−14	4.35E−14
^181^Hf	2.34E−12	2.11E−12	1.97E−12	1.87E−12	1.73E−12	1.50E−12	1.25E−12	1.09E−12	8.89E−13	6.31E−13	4.09E−13	3.04E−13	2.02E−13	1.10E−13
^182^Ta	5.34E−12	4.83E−12	4.51E−12	4.28E−12	3.96E−12	3.45E−12	2.89E−12	2.54E−12	2.10E−12	1.52E−12	1.02E−12	7.73E−13	5.23E−13	2.91E−13
^192^Ir	3.61E−12	3.27E−12	3.05E−12	2.90E−12	2.68E−12	2.34E−12	1.95E−12	1.71E−12	1.40E−12	9.98E−13	6.46E−13	4.80E−13	3.19E−13	1.74E−13
^203^Hg	1.04E−12	9.43E−13	8.82E−13	8.38E−13	7.76E−13	6.75E−13	5.62E−13	4.91E−13	4.00E−13	2.83E−13	1.80E−13	1.33E−13	8.78E−14	4.76E−14
^214^Pb	1.11E−12	1.00E−12	9.35E−13	8.88E−13	8.21E−13	7.14E−13	5.92E−13	5.19E−13	4.22E−13	2.99E−13	1.92E−13	1.42E−13	9.42E−14	5.12E−14
^214^Bi	5.93E−12	5.37E−12	5.03E−12	4.78E−12	4.43E−12	3.88E−12	3.26E−12	2.88E−12	2.40E−12	1.76E−12	1.18E−12	9.02E−13	6.16E−13	3.45E−13
^222^Rn	1.72E−15	1.55E−15	1.45E−15	1.38E−15	1.27E−15	1.11E−15	9.26E−16	8.14E−16	6.69E−16	4.79E−16	3.14E−16	2.35E−16	1.57E−16	8.59E−17
^239^Np	7.55E−13	6.73E−13	6.26E−13	5.92E−13	5.45E−13	4.70E−13	3.87E−13	3.35E−13	2.69E−13	1.85E−13	1.15E−13	8.41E−14	5.49E−14	2.94E−14
^238^Pu	4.69E−15	2.37E−15	1.59E−15	1.21E−15	8.29E−16	4.74E−16	2.64E−16	1.85E−16	1.17E−16	6.21E−17	3.24E−17	2.20E−17	1.34E−17	6.79E−18
^239^Pu	2.27E−15	1.25E−15	8.97E−16	7.23E−16	5.43E−16	3.63E−16	2.42E−16	1.90E−16	1.38E−16	8.62E−17	5.11E−17	3.65E−17	2.34E−17	1.24E−17
^240^Pu	4.44E−15	2.25E−15	1.51E−15	1.15E−15	7.95E−16	4.57E−16	2.56E−16	1.80E−16	1.15E−16	6.13E−17	3.22E−17	2.19E−17	1.34E−17	6.78E−18
^241^Pu	6.71E−18	5.84E−18	5.38E−18	5.07E−18	4.63E−18	3.96E−18	3.21E−18	2.75E−18	2.16E−18	1.44E−18	8.72E−19	6.25E−19	4.02E−19	2.12E−19
^241^Am	1.31E−13	1.02E−13	8.89E−14	8.09E−14	7.02E−14	5.54E−14	4.05E−14	3.25E−14	2.36E−14	1.41E−14	7.88E−15	5.47E−15	3.39E−15	1.75E−15
^242^Cm	5.27E−15	2.72E−15	1.85E−15	1.41E−15	9.72E−16	5.55E−16	3.07E−16	2.14E−16	1.34E−16	7.07E−17	3.68E−17	2.49E−17	1.52E−17	7.71E−18

^214^Bi contributes most significantly to the photon field in air, among the decay products of ^238^U. If the ^238^U decay products are in radiation equilibrium in ground, the kerma in air due to ^214^Bi contributes nearly 90 % to the total kerma due to the nuclides in the ^238^U series (Saito and Jacob 1995, 1998). Further, when ^214^Bi, produced by decay of ^222^Rn in air, is deposited on ground by rain, it significantly contributes to an increase in kerma rate above ground. The water containing ^214^Bi could then migrate into soil and form a volume source within a short period of time; therefore, the data shown here could be used to evaluate the increase in air kerma rate due to precipitation, because the coefficients are shown here for a wide range of relaxation depth up to 100 g/cm^2^ which is close to a volume source.

In Fig. 2, illustrations are shown for radionuclides plotted in the order of increasing emitted photon energy. It can be seen that, for ^151^Sm, the effective dose for Baby is three times higher than for adults, since the emitted photon energies are below 20 keV. The difference in effective dose between Baby and adults becomes smaller for high-energy photons. In case of ^241^Am, which emits mainly 60 keV photons, the difference is 70–90 %; while, for ^137m^Ba that is a decay product of ^137^Cs, for ^60^Co and for ^214^Bi, emitting higher-energy photons, the difference in effective dose with body size is around 30 %.

Saito and coworkers (Saito et al. 1991) performed a comprehensive investigation of the effect of body size on organ and effective doses, for mono-energetic environmental sources. It was concluded that, above 50 keV source energy, the maximum difference between Baby and adults is less than a factor of 2 for effective doses and less than a factor of 3 for individual organ doses. This finding is in accordance with the results shown here.

The dose coefficient decreases rapidly with relaxation depth, due to the large shielding effect of soil for low-energy photons. As the photon energy increases, the shielding effect by soil decreases. The difference in effective dose between relaxation depths of 0 and 5 g/cm^2^ varies between factors of 2–5, except for ^151^Sm.

### Statistical analysis of effect of body size

**Fig. 3:**
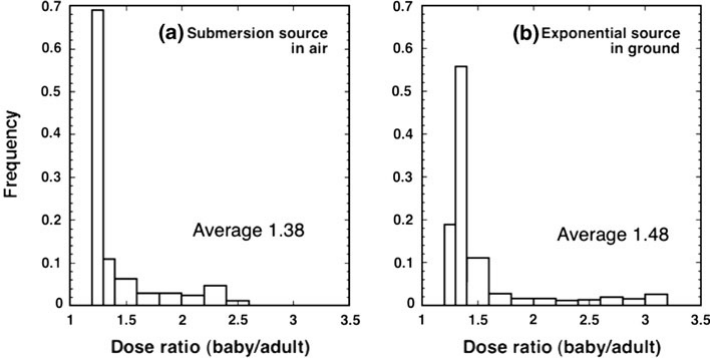
Distribution of effective dose ratios between Baby and adults for **a** a submersion source in air and **b** an exponential source in ground. The dose coefficients for the submersion source in air were taken from Petoussi-Henss et al. 2008

In the present study, a statistical analysis was performed concerning the ratio of effective doses for Baby and adults for radionuclides. Frequency distributions of the ratios for the 185 radionuclides for which conversion coefficients were computed are shown in Fig. 3 (right). For comparison, Fig. 3 (left) shows frequency distributions for conversion coefficients for submersion in a radioactive cloud, that is, in volume source in air obtained earlier by the authors (Petoussi-Henss and Saito 2009; Peoussi-Henss et al. 2008).

It can be seen that the differences are greater for the exponential sources in ground: For submersion sources, the maximum ratio is about 2.5, while it is about 3 for the exponential ground source. The average ratio is 1.4 and 1.5, respectively. This is because the radiation fields for the exponentially distributed source in the ground clearly change with height above ground. For example, the air kerma at 1 m height from an exponentially distributed source with relaxation of 0.1 g/cm^2^ is smaller by 20 % than that at 0.1 m height. This reduction of air kerma with height above ground is reflected in effective doses calculated on the assumption that the human phantoms used here (Baby and adults) stand right up on the ground; several important organs of the adult and Baby phantoms are positioned then at different heights.

It must be noted that in more than 80 % of the cases, the difference in effective doses due to body size is less than 50 %. Radionuclides showing large discrepancies are mostly transuranium nuclides which do not contribute much to external exposures. A similar tendency was observed when the statistical analysis was performed after selecting those 24 radionuclides reported to be greatly released during the Chernobyl accident. Therefore, it can be assumed that, using the effective dose conversion coefficients for reference adults, one can generally evaluate effective doses in the environment for people of various ages and statures within 50 % uncertainty. Similarly, the coefficients for Baby can be used to estimate effective doses for infants. If more suitable doses for different ages are necessary, further simulations using additional phantoms are needed.

### Comparison between effective doses calculated based on ICRP Publications 103 and 60

**Fig. 4:**
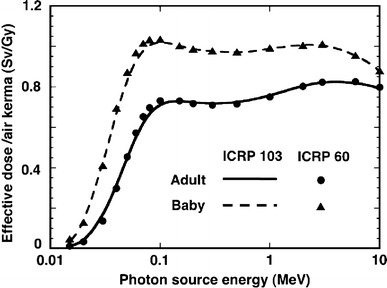
Comparison of effective doses defined in ICRP 103 to those defined in ICRP 60, for a plane source at a depth of 0.5 g/cm^2^ in ground. The data for ICRP 103 were taken from Petoussi-Henss et al. 2008

Figure 4 compares the effective dose conversion coefficients for a mono-energetic plane source at a depth of 0.5 g/cm^2^ in ground, as calculated following the definitions of ICRP Publication 60 (ICRP 1991) and 103 (ICRP 2007), for both adults and the Baby. It can be seen that the variations introduced by implementation of different tissue weighting factors are quite small for both anthropomorphic phantoms.

Generally, change in the tissue weighing factors does not significantly influence the effective dose for environmental external exposure. In this case, the incident angle distribution of photons is assumed symmetrical around the body axis; then, any difference in weighted organ doses tends to be smeared by averaging around the body axis. It should be noted that the difference between the effective dose equivalent as defined in ICRP Publication 26 (ICRP 1977) and the effective dose of ICRP Publication 60 (ICRP 1991) was found also not to be significant for environmental photon exposures (Saito et al. 1998).

Furthermore, the effective dose conversion coefficients for radionuclides were also calculated according to the ICRP 60 definition and compared with those for the ICRP 103 (ICRP 2007) definition for all 185 nuclides. The difference was found to be within 10 %, and about 80 % of the coefficients agreed within a few per cent. When the conversion coefficients calculated using the stylized phantoms Adam and Eva according to the ICRP 60 definition were considered, the discrepancies became a little larger; nevertheless, the maximum difference is still within 10 %. Thus, the effective dose conversion coefficients shown in the present paper can be used for protection systems implementing the definitions of ICRP Publication 60 (ICRP 1991) with negligible errors.

## Conclusion

Effective dose conversion coefficients, that is, the effective dose rate per unit activity per unit area, were computed for sources exponentially distributed in the ground, based on reliable methodologies previously developed. The data were evaluated for various relaxation depths of a source exponentially distributed in ground, including a variety of radionuclides, which could potentially contaminate the ground after accidental radioactive releases, as well as some natural radionuclides. It was shown that these data could apply to evaluate environmental exposures under various conditions. Generally, effective dose conversion coefficients at relaxation depth of 5 g/cm^2^ are smaller by a factor of 2–5 than those at 0 g/cm^2^ mainly because of the shielding effect by soil. In order to evaluate the effective doses for infants, the conversion coefficients for an 8-week-old phantom of a Baby were evaluated. Using these data, the difference in effective dose due to body size was found, for most cases, to be within 50 %; for some radionuclides emitting low-energy photons, however, the difference could amount to a factor of 2–3. It was also shown that the effective dose conversion coefficients for radionuclides investigated in the present paper are in accordance with both definitions, as described in ICRP Publication 60 (ICRP 1991) (which represents the basis for national laws on radiation protection in many countries up to now) and in the latest recommendations of ICRP Publication 103 (ICRP 2007).
